# Sigmoid mesocolon internal hernia: a case report

**DOI:** 10.1097/MS9.0000000000000566

**Published:** 2023-04-06

**Authors:** Kiki Lukman, Bambang Am Am Setya Sulthana, Rio Andreas, Prapanca Nugraha

**Affiliations:** Division of Digestive Surgery, Department of Surgery, Faculty of Medicine, Padjadjaran University/Dr. Hasan Sadikin General Hospital, Bandung, Indonesia

**Keywords:** case report, CT scan, hernia, internal hernia

## Abstract

Primary internal hernias are rare in adults. Internal hernias present clinically as a sign of small intestinal obstruction. If left untreated, internal hernias might lead to high morbidity and mortality due to strangulation. Internal hernias are usually diagnosed intraoperatively. Here, we present an internal hernia diagnosed with an abdominal computed tomography (CT) scan. The importance of preoperative diagnosis for internal hernias leads to early surgery and keeps the patient from suffering intestinal strangulation.

**Case presentation:**

We report here the case of a 67-year-old male who presented with acute intestinal obstruction and underwent an abdominal CT scan. The patient was diagnosed with an internal hernia from the imaging of the abdominal CT scan and scheduled for an exploratory laparotomy. An internal hernia was found in the mesocolon of the sigmoid colon; one loop of jejunum was trapped in the hernia defect. After reduction, the hernial defect was closed; no resections were done, and the patient was discharged after 5 days without complication.

**Clinical discussion:**

Our finding represents a transmesosigmoid hernia, a rare variant of sigmoid mesocolon hernias. The clinical sign and the judgment of the surgeon for the diagnosis of an internal hernia became important factors related to the patient’s outcome.

**Conclusion:**

The proper adjunct imaging, correct diagnosis, and timing of surgery for internal hernias save the patient from morbidity or intestinal death.

## Introduction

Highlights

- Internal hernias are rare.
- Transmesosigmoid hernia is one of the rarest types of internal hernias.
- This condition can lead to strangulation or incarceration of the intestines.
- The right imaging, diagnosis, and timing of surgery can decrease morbidity.

An ‘internal hernia’ is a protrusion of the small intestine or another abdominal organ through a mesenteric defect or peritoneal orifices within the peritoneal cavity. This condition can occasionally result in strangulation or incarceration of the intestines^1^–^3^. Internal hernias account for only 0.2–0.9% of cases of intestinal obstruction. Internal hernias have been reported to have an overall mortality of more than 50% without surgery^4^. Sigmoid mesocolon hernia is a rare form of congenital internal hernia, and transmesosigmoid hernia is one type of sigmoid mesocolon hernia^5^. Internal hernias are difficult to clinically diagnose because of their vague signs and symptoms. For proper care, a preoperative diagnosis must be accurate. A computed tomography (CT) scan is an important part of the preoperative diagnosis of internal hernias. A CT scan can reveal an intestinal obstruction, the hernia orifice and sac, intestine dilatation, collapse, or intestinal twisting^6^. Clinical presentation of the internal hernia is associated with obstruction symptoms such as abdominal pain, nausea, vomiting, constipation, and distention. Hence, internal hernias are usually detected as an intraoperative finding in emergency laparotomies^5^,^6^. A preoperative diagnosis is rarely confirmed in an emergency setting. In this report, we describe an intramesosigmoid hernia that was diagnosed by an abdominal CT finding.

## Case report

**Figure 1:**
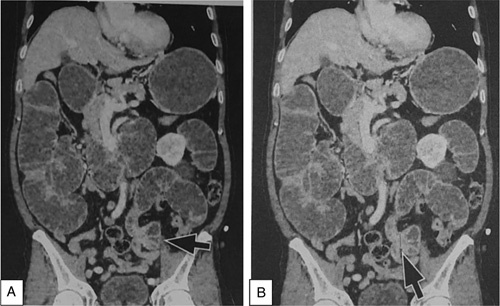
Abdominal computed tomography scan showing an internal hernia. (A) Intramesosigmoid hernia at the distal jejunum (arrow). (B) Neck of intramesosigmoid hernia (arrow).

**Figure 2:**
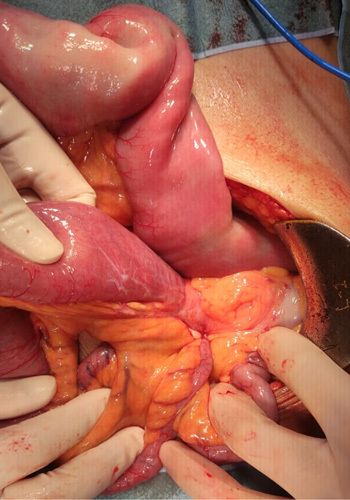
Intraoperative finding of trapped jejunum in the mesocolon sigmoid defect.

**Figure 3:**
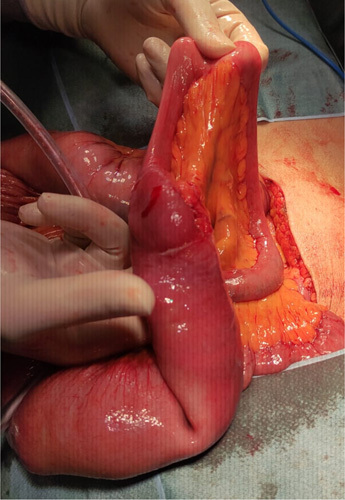
Incarcerated intestines after reduction: the proximal jejunum was dilated and the distal jejunum was collapsed.

**Figure 4:**
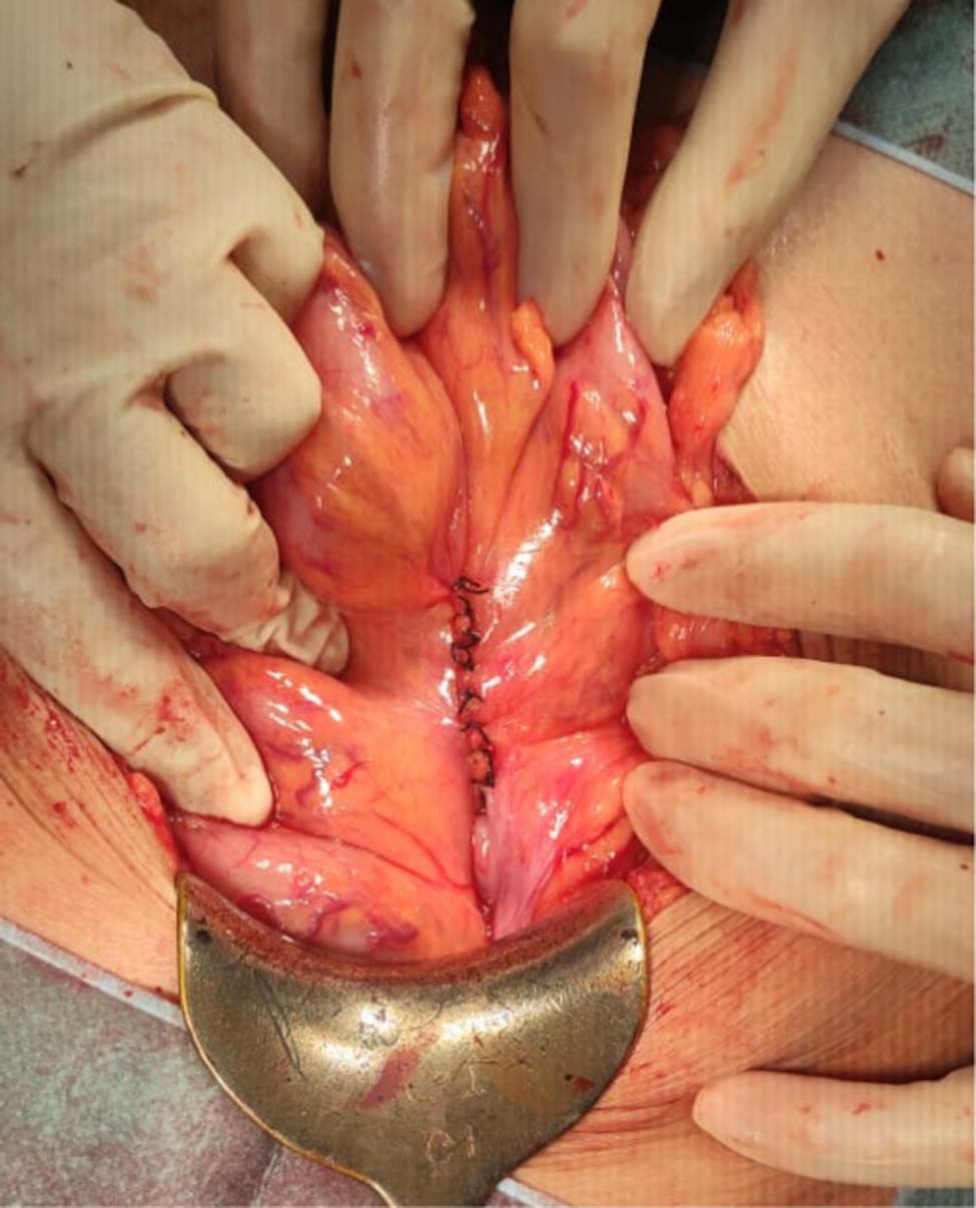
A hernial defect in the mesocolon sigmoid was repaired with interrupted nonabsorbable sutures.

The SCARE criteria were followed in reporting this case report^7^. A 67-year-old Asian male presented to the emergency department with a chief complaint of vomiting. He had been suffering from a bloated and enlarged stomach for 1 week, and he had been vomiting, primarily after meals, for 2 days before his admission. He had also been unable to defecate for the previous 3 days. He had no prior history of surgical procedures previously or comorbidities. He had no history of chronic medication or allergies previously. Physical examination revealed tachycardia and moderate abdominal pain; other physical examination findings were within normal ranges. After fluid rehydration, he was taken for an abdominal CT scan. The abdominal CT scan showed a herniation of the distal jejunum measuring 6.1 cm long, located in the sigmoid mesocolon with a neck width of 2.01 cm, the proximal part of the intestine looking dilated, without wall thickening and the distal part of the intestine looking collapsed (Fig. 1). The patient was scheduled for surgery and underwent an exploratory laparotomy. The surgery was performed by one consultant gastrointestinal surgeon, one gastrointestinal fellow, and one surgical resident in district general hospital. Intraoperatively, a loop of jejunum was found to enter the mesenteric colon at the sigmoid colon through a defect with a diameter of 2.5 cm (Fig. 2). The proximal jejunum from the hernia defect was dilated and the diameter enlarged in contrast to the distal jejunum from the hernia defect, which seemed to reduce in size (Fig. 3). The ileum was found to be viable after reduction and compression with warm saline for 15 min, there was no need for resection of the intestines. The hernia defect was repaired with nonabsorbable interrupted sutures (Fig. 4). There was no strangulation or malrotation of the intestines. The patient’s recovery went without any complications, and he was discharged on the fifth postoperative day. There are no complication signs of bloating, abdominal pain, recurrence, or infection at the surgical site^5^,^8^. He was advised to follow a soft diet for a week and a normal diet after. One month after surgery, he went for a follow-up and was doing well without any symptoms or complications.

## Discussion

Internal hernias account for less than 1% of cases of small bowel obstruction, with the underlying causes being primarily peritoneum or mesenteric defects^8^,^9^ or prior history of surgical procedure^10^–^12^. Clinical symptoms of internal hernias are nonspecific and include intermittent abdominal pain, distension, nausea, and vomiting, and the patient might be presenting with partial or complete acute intestinal obstruction^6^,^13^–^16^.

Investigations for the diagnosis of an internal hernia are a plain abdominal radiograph, a barium examination, and an abdominal CT scan. Despite the fact that an abdominal CT scan has a sensitivity and specificity of 63 and 73%, respectively, in diagnosing an internal hernia, internal hernias are frequently diagnosed intraoperatively in patients with acute intestinal obstruction during emergency laparotomies. The management of intramesosigmoid hernias includes reduction of the hernia and repair of the defect, which can either be accomplished laparoscopically or by an open approach^17^–^19^. Open approaches offer good ergonomics for the surgeons and mobility for the equipment, but laparoscopic procedures offer advantages of fewer complications and a shorter hospital stay^20^–^24^.

Benson and Killen^25^, in 1964, classified sigmoid mesocolon hernias into the following three types:

1. Intersigmoid hernia: the herniation at the point where the lateral side of the sigmoid mesocolon attaches, there is a herniation into the inter-sigmoid fossa. This fossa is created when the parietal peritoneum of the posterior abdominal wall and the left peritoneal surface of the sigmoid mesentery fuse.
2. Transmesosigmoid hernia: the intestinal loops are incarcerated because of a full-thickness sigmoid mesocolon defect.
3. Intramesosigmoid hernia: The sigmoid mesocolon has one leaf (lateral is more common), which is affected by a congenital, oval defect next to the colon that is unrelated to the intersigmoid fossa and causes herniation.

**Figure 5:**
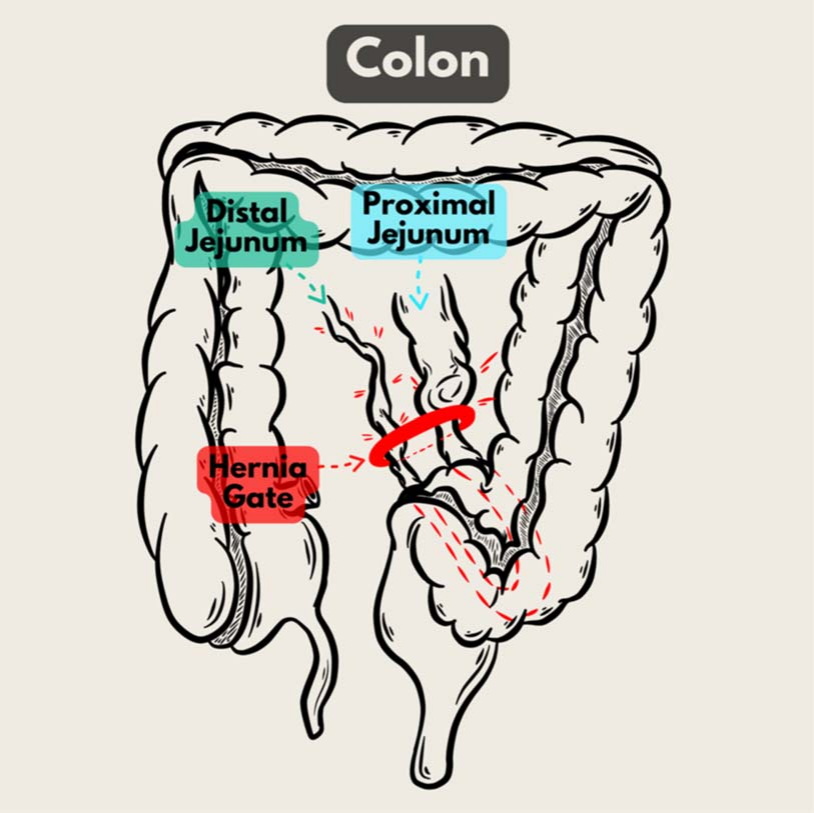
A schematic that shows the intramesosigmoid hernia in our case: a hernia gate located at the sigmoid mesocolon, with a dilated proximal jejunum and a collapsed distal jejunum.

The internal hernia in this report is a transmesosigmoid hernia because a loop of jejunum was trapped and incarcerated in a sigmoid mesocolon full-thickness defect (Fig. 5). Internal hernias might lead to intestinal strangulation, which puts the intestines at risk for gangrene and perforation^2^,^5^. A rare case of internal hernia made the proper imaging and timing of the surgery important to prevent intestinal strangulation. The timing of diagnosis and surgical management will still be important factors for patient morbidity and mortality.

## Conclusions

Internal hernias are not very common, and sigmoid hernias are one of the rarest among internal hernias. One type of internal sigmoid hernia is a transmesosigmoid hernia. Internal hernias are frequently difficult to diagnose based solely on an abdominal radiograph, which can show signs of intestinal obstruction. The proper adjunct imaging, correct diagnosis, and timing of surgery for internal hernias save the patient from morbidity or intestinal death.

## Ethical approval

No ethical approval was necessary.

## Consent

The patient’s written informed consent was obtained before this case report and the associated pictures could be published. The Editor-in-Chief of this journal can review a copy of the written consent upon request.

## Sources of funding

The author(s) received no financial support for this case report.

## Author contribution

All the authors participated in drafting the manuscript and finalizing it.

## Conflicts of interest disclosure

The authors declare that they have no financial conflict of interest with regard to the content of this report.

## Research registration unique identifying number (UIN)

Not applicable.

## Guarantor

Kiki Lukman.

## Provenance and peer review

Not commissioned, externally peer-reviewed.
